# Precipitation Behavior during Aging Operations in an Ultrafine-Grained Al–Cu–Mg Alloy Produced by High-Strain-Rate Processing

**DOI:** 10.3390/ma15238687

**Published:** 2022-12-06

**Authors:** Linyan Zhang, Hongyun Luo

**Affiliations:** 1 School of Materials Science and Engineering, Beihang University, Beijing 100191, China; 2 Beijing Key Laboratory of Advanced Nuclear Materials and Physics, Beihang University, Beijing 100191, China

**Keywords:** Al–Cu–Mg alloy, grain boundary, precipitation behavior, elements segregation, high strain rate

## Abstract

An ultrafine-grained (UFG) Al–Cu–Mg alloy (AA2024) was produced by surface mechanical grinding treatment (SMGT) with a high strain rate, and the precipitation behavior inside the grain and at the grain boundary was investigated. During SMGT, element segregation at the boundary was rarely observed, since the solute atoms were impeded by dislocations produced during SMGT. During early aging, the atomic fraction of Cu at the grain boundary with SMGT alloys was approximately 2.4-fold larger than that without SMGT alloys, the diffusion rate of Cu atoms from the grain toward the grain boundaries was accelerated with SMGT alloys, because a higher local elastic stress and diffusion path were provided by high-density dislocations. The combined action, in terms of the composition of the alloy, the atomic radius, the diffusion path, and the diffusion driving force provided by high-density dislocations with SMGT alloys, led to a Cu/Mg atomic ratio of approximately 6.8 at the grain boundary. The average size of the precipitates inside the grain was approximately 2- and 10-fold larger than that formed after later aging with and without SMGT alloys, due to more nucleation sites at dislocation located inside the grain with SMGT alloys having attracted and captured numerous solute atoms during the aging process.

## 1. Introduction

Al–Cu–Mg alloys are among the most important age-hardenable aluminum alloys and are intensively applied in the fields of architecture and aerospace, where good specific strength is required [1,2]. The properties of these age-hardenable alloys, such as toughness and susceptibility to yield strength, are mainly affected by the distribution of precipitates that are formed in the matrix during the aging process. In order to improve these properties, the optimization of the precipitation behavior of these alloys has attracted much attention [3,4,5,6,7,8,9,10,11,12,13,14,15,16,17,18,19,20,21,22,23]. Furthermore, severe plastic deformation (SPD) techniques with different strain rates have also been widely applied to change the microstructure of aluminum alloys in order to strengthen them [24,25,26,27,28,29,30,31,32].

SPD has a great influence on the precipitation behavior of age-hardenable aluminum alloys during the aging process [28,33,34,35,36,37,38,39]. Most of the existing studies mainly focus on precipitation behavior in UFG alloys produced by SPD with a lower strain rate (<10 S^−1^), including characterization of element segregation [24,27,40], precipitation at the grain boundary [26,27,28,41], assessment of the precipitate size [4,41], and analysis of the precipitation kinetics [34,38,42] or the precipitation sequence [28,42,43]. Moreover, the grain boundary has been reported to significantly affect the element segregation and precipitation behavior of alloys. Cu [25,44] and Mg [45] element segregation along the boundary during SPD (before aging) with a lower strain rate has been reported. Moreover, for UFG alloys produced by SPD with a lower strain rate, during the aging process, investigation has indicated that the ratio of Cu and Mg atoms at the grain boundary is close to 1, and that the concentration of Mg is higher than that of Cu near the grain boundary in UFG Al–Cu–Mg alloys [24]. Jia et al. [27] reported that Cu element segregation at the grain boundary is promoted in UFG Al–5Cu alloys. The formation of precipitates at the grain boundary is also facilitated [26,27] and the thermal stability of precipitates is higher in UFG alloys induced by SPD with a lower strain rate compared to without SPD alloys [41,46]. Even though the results of such studies can serve as a reference for determining the precipitation behavior in UFG alloys, for an alloy produced with different strain rates, the grain size is possibly decreased [47] and the dislocation density increased [31]. Furthermore, grain boundary relaxation may occur [48], which could result in different precipitation behaviors. Therefore, it is crucial to develop a deep understanding of the mechanisms that govern the precipitation behavior in age-hardenable UFG alloys produced by SPD with a high strain rate (10^3^~10^5^ S^−1^) [31]. 

SMGT is a type of SPD with a high strain rate and has been successfully applied to produce nano-laminated surface layers on various materials. SMGT is an efficient method used to improve the mechanical properties [49,50], fatigue, and wear resistance of metallic materials [51] and achieves this by optimizing the processing parameters, such as sliding velocity and penetration depth. Therefore, SMGT was selected as the processing method in this work. 

In this paper, the precipitation behavior of UFG alloys produced by SMGT was investigated during the aging process. An Al–Cu–Mg alloy was chosen as the experimental material and was subjected to SMGT at room temperature, as well as aging treatments at intermediate temperatures for different durations of time. The morphology, composition, and distribution of the precipitates, as well as their thermal stability with SMGT alloys, were analyzed. Such a systematic investigation provides new insights into the precipitation behavior of UFG Al–Cu–Mg alloys, which could improve the mechanical performance of alloys.

## 2. Experimental Procedures

### 2.1. Materials and Processing 

**Figure 1:**
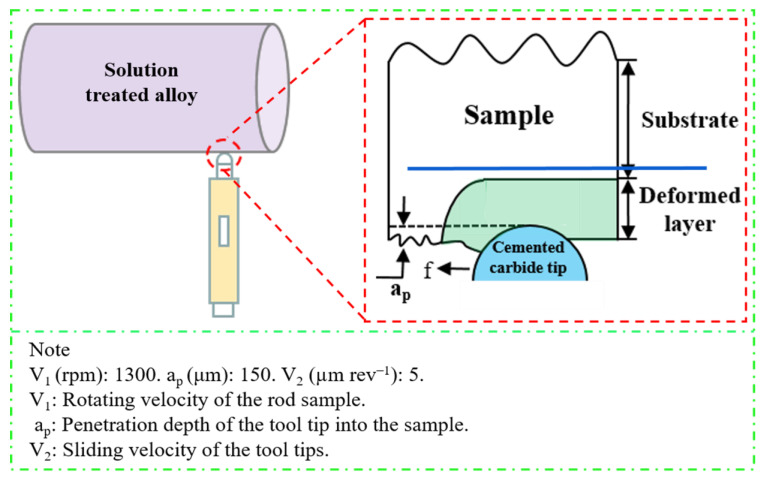
Schematic of the SMGT.

A commercially available 2024 aluminum alloy rod mainly composed of Al-4.3 Cu-1.5 Mg and small amounts of Si, Mn, Fe, Zn, Cr, and Ti (wt.%) was used in this work. A cylindrical bar with a diameter of 90 mm and a length of 200 mm was subjected to solution treatment at 495 °C for 10 h, followed by water quenching to obtain a supersaturated solid solution. Then, SMGT with a polycrystalline diamond (PCD) tool (with a radius of r = 1.5 mm) was applied at room temperature, using a precision lathe (CM0420M/2). A schematic of this is shown in Figure 1. The SMGT process was repeated twice with the same processing parameters to obtain a thick deformation layer. Subsequently, Al sheets were cut with a wire-cutting machine from the topmost layer of 0.3 mm in the perpendicular direction to rotation. The solution treated without SMGT alloys was used as a comparison.

Next, the samples were aged at 210 °C for different durations of time (6, 27, and 60 min) and cooled to room temperature in a furnace at 0.1 °C/s. Then, the SMGT alloy was polished with waterproof abrasive paper (200–2000#) from the depth layer to the surface layer, and a thickness of 70 μm was selected, as well as without the SMGT alloy, polished from two sides of a sample to 70 μm. 

### 2.2. Microstructure Characterization 

The samples were prepared using twin-jet electropolishing from the depth layer to the surface layer in a mixture of 70% methanol and 30% nitric acid at 30 °C and 20 V. Five samples were prepared under each condition. Bright-field images and high-resolution transmission electron microscope (HRTEM) images of the SMGT Al-2024 and the solution-treated samples were characterized using JEOL 2100 F transmission electron microscopy operating at an accelerating voltage of 200 kV. Energy-dispersive X-ray spectroscopy (EDS) analysis of elements was performed on a high-angle annular dark-field scanning transmission electron microscope (HAADF-STEM) using the same sample in the case of HRTEM. Wide-angle X-ray scattering measurements were performed on the 1W2A beamline of the Beijing Synchrotron Radiation Facility (BSRF, Beijing, China). The sample produced was a UFG alloy, the X-ray wavelength was 1.54 Å, and the beam size was 1 × 0.4 mm^2^.

The precipitate sizes following aging with and without SMGT samples were obtained using bright-field TEM and HRTEM observations of over 1800 precipitates in 1000 grains. 

## 3. Results and Analysis

### 3.1. Microstructure in the Initial State

**Figure 2:**
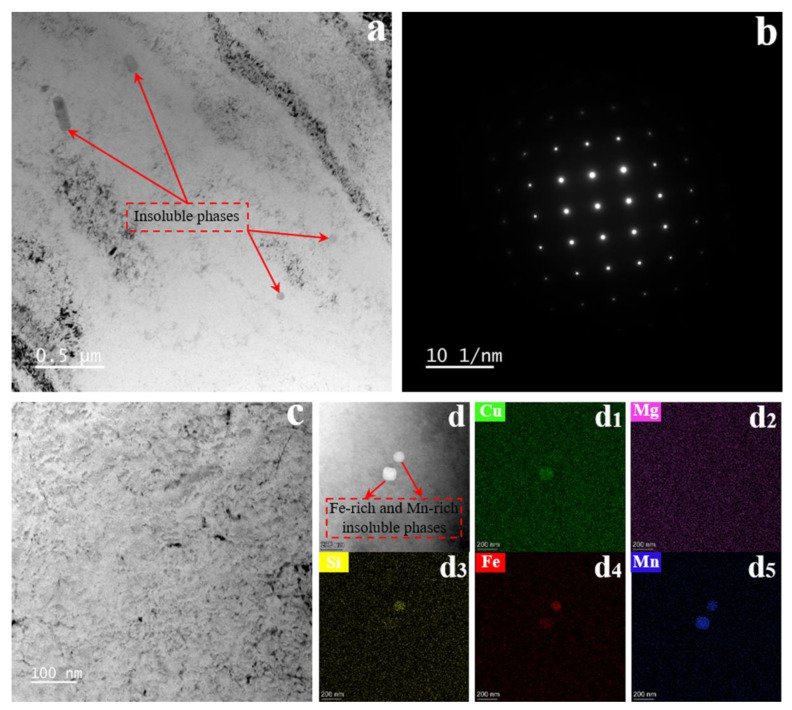
The solution treated without SMGT alloys. Low-magnification bright-field TEM image (**a**) and the corresponding selected area diffraction pattern (**b**), high-magnification bright-field TEM image (**c**), HAADF-STEM image (**d**), and the corresponding EDS elemental maps of copper (**d_1_**), magnesium (**d_2_**), silicon (**d_3_**), iron (**d_4_**), and manganese (**d_5_**).

Figure 2 illustrates the solution treated without SMGT alloys. The representative bright-field TEM images in Figure 2a reveal a microstructure consisting of an inhomogeneous distribution of trace coarse insoluble impurity phases. The corresponding selected area diffraction pattern (SAD) shows few diffuse streaks of the precipitates, indicating that precipitates were not produced (Figure 2b). Moreover, no precipitates were observed in the grain interiors shown in the high-magnification bright-field TEM images (Figure 2c). To further identify the element distribution in the solution treated without SMGT alloys, HAADF-STEM images (Figure 2d) and corresponding EDS maps (Figure 2d_1_–d_5_) were collected. The results indicate that the elements were uniformly distributed in the alloy, except for the coarse insoluble impurity phases containing Cu, Si, Fe, and Mn.

**Figure 3:**
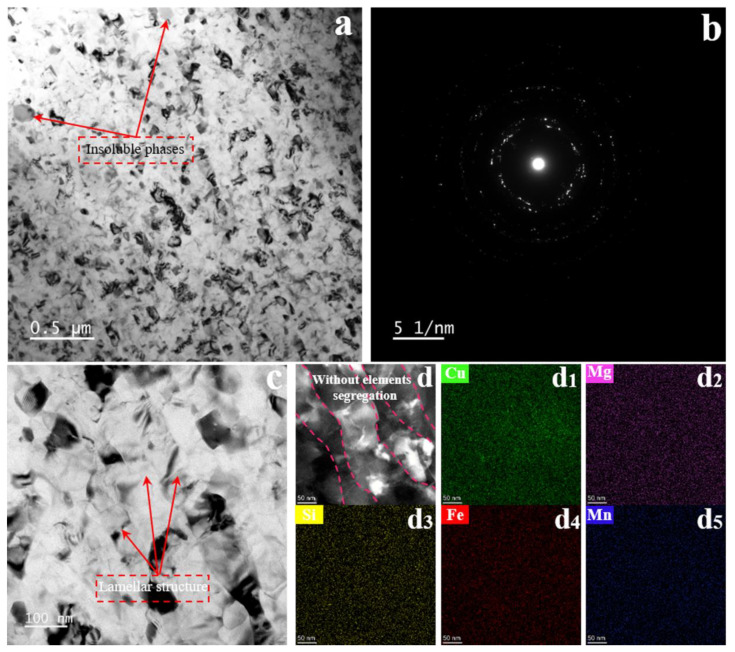
The solution treated with SMGT alloys. Low-magnification bright-field TEM image (**a**) and the corresponding selected area diffraction pattern (**b**), high-magnification bright-field TEM image (**c**), HAADF-STEM image (**d**), and the corresponding EDS elemental maps of copper (**d_1_**), magnesium (**d_2_**), silicon (**d_3_**), iron (**d_4_**), and manganese (**d_5_**).

The solution treated with SMGT alloys is shown in Figure 3. After the treatment, in the surface layer, elongated grains with an average transversal size of 53.3 nm and an aspect ratio of 2.6 were formed, and coarse insoluble impurity phases existed (Figure 3a). The STEM-EDS results (Supplementary Figure S1) reveal these coarse insoluble impurity phases containing Cu, Si, Fe, and Mn, suggesting that an Fe, Mn-rich phase was formed, which is consistent with the results observed in the solution treated without SMGT alloys (Figure 2). Additionally, the corresponding SAD pattern in Figure 3b exhibits the approximate ring, which shows that the solution treated with SMGT alloys was composed of numerous approximate nanometer grains.

Figure 3c,d, respectively, display high-magnification bright-field TEM images and HAAD-STEM images of a lamellar structure with the central grain orientated parallel to the shear direction. It can be seen that the element distribution was uniform in the alloy and segregation along the grain boundaries was rarely observed, as shown in the corresponding EDS elemental maps (Figure 3d_1_–d_5_). This phenomenon is different to some previous reports, which revealed Cu [25,44] and Mg [45] element segregation along the boundary during the SPD process.

### 3.2. Precipitation with and without SMGT Alloys

**Figure 4:**
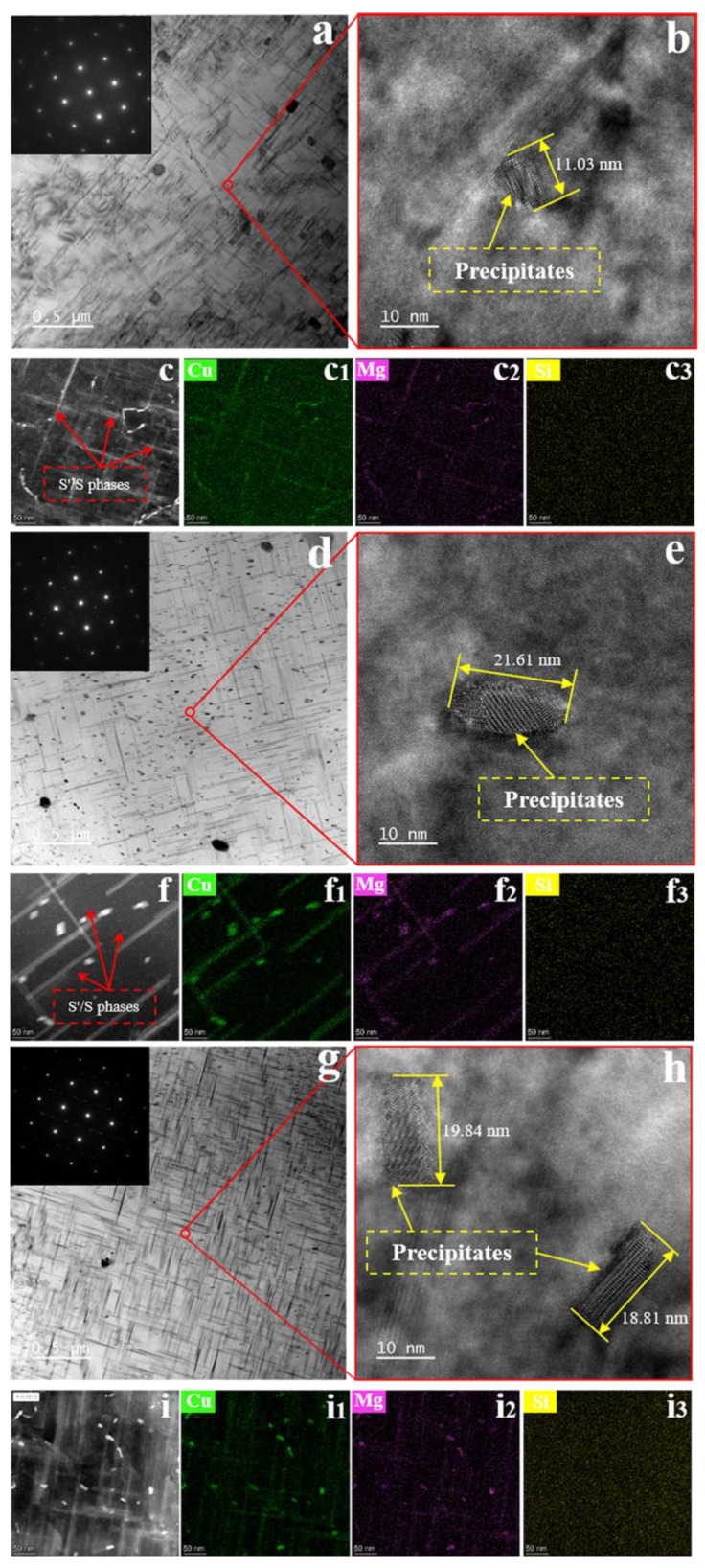
Microstructure without SMGT alloys aged at 210 °C for different times. Bright-field TEM images with an inset of the representative selected area diffraction pattern: (**a**) 6 min, (**d**) 27 min, and (**g**) 60 min. The corresponding high-resolution TEM images are indicated in (**b**,**e**,**h**), respectively. The size of the precipitate is marked in the high-resolution TEM images. HAADF-STEM images of precipitate region are displayed in (**c**,**f**,**i**), respectively. The corresponding EDS elemental mappings are shown in (**c_1_**–**c_3_**), (**f_1_**–**f_3_**), and (**i_1_**–**i_3_**), respectively.

The microstructure without SMGT alloys aged at 210 °C for different times is presented in Figure 4. Bright-field TEM images of the without SMGT alloys aged for 6, 27, and 60 min are shown in Figure 4a,d,g, respectively, revealing that the microstructure consisted of the uniform distribution of precipitates that were needle-like and rod-shaped. It can be seen that more needle-like and rod-shaped precipitates were produced with increased aging time. Particularly for the needle-like precipitates, it can be seen that there was significant growth after a longer aging time.

Moreover, a significant increase in intensity of the precipitate spots was observed in the SAD patterns with aging time, indicating that a further nucleation precipitation and coarsening process occurred inside the grain with increasing aging time. The corresponding HRTEM micrographs of the microstructure aged for 6, 27, and 60 min are shown in Figure 4b,e,h, respectively. The lengths of the precipitates were approximately 11, 21, and 19 nm, respectively, with aging time, confirming the formation of nanoscale precipitates in the alloy. The STEM-EDS pattern results revealed these needle-like and rod-shape precipitates distributed in the bulk matrix to be Cu- and Mg-rich, suggesting that numerous S′/S phases were formed during aging process, as shown in Figure 4c–c_3_,f–f_3_,i–i_3_. The above results obtained without SMGT alloys show that S′/S phases were formed inside the grain during the aging process, and more nucleation and growth occurred with increasing aging time. 

**Figure 5:**
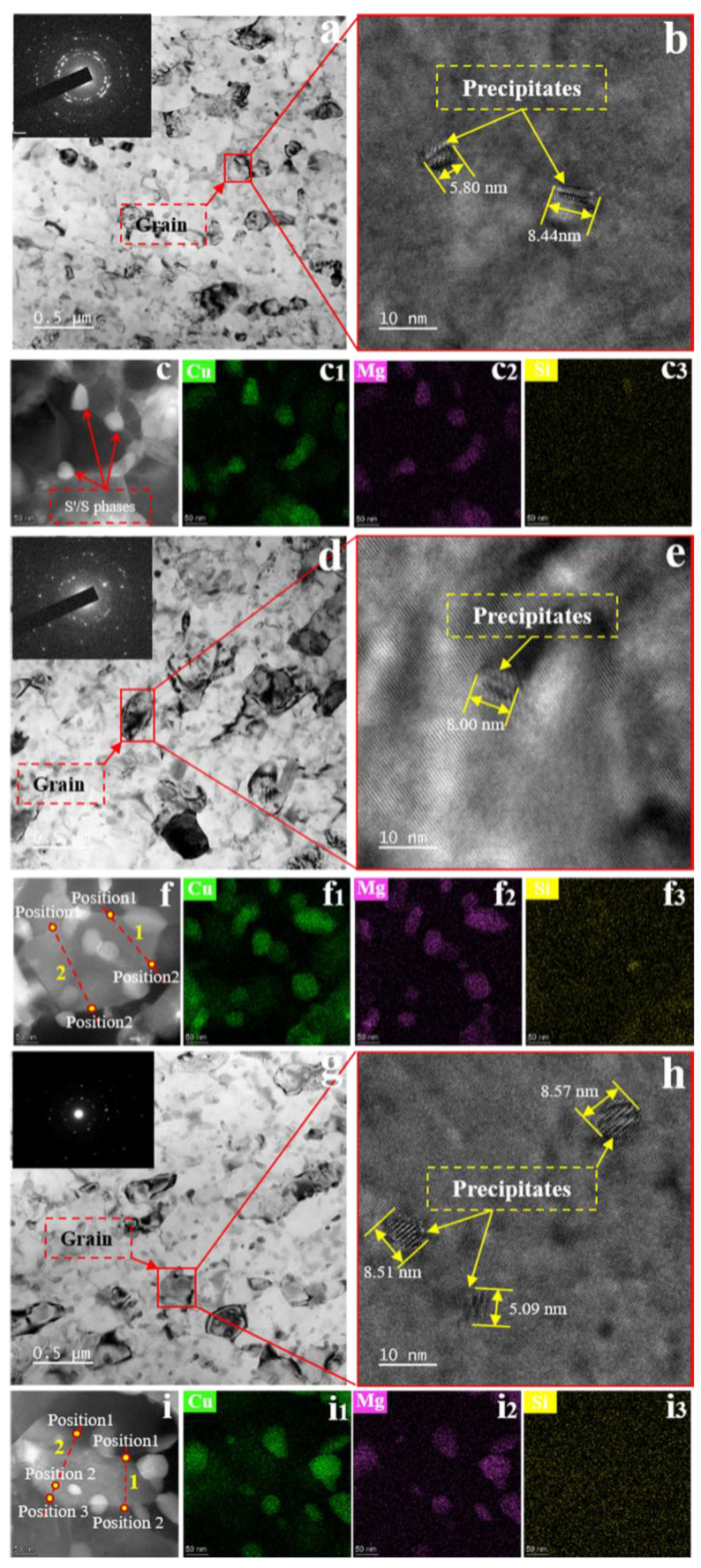
Microstructure with SMGT alloys aged at 210 °C for different times. Bright-field TEM images with an inset of the representative selected area diffraction pattern: (**a**) 6 min, (**d**) 27 min, and (**g**) 60 min. The corresponding high-resolution TEM images are indicated in (**b**,**e**,**h**), respectively. The size of the precipitate is marked in the high-resolution TEM images. HAADF-STEM images of the precipitate region are displayed in (**c**,**f**,**i**), respectively. The corresponding EDS elemental mappings are shown in (**c_1_**–**c_3_**), (**f_1_**–**f_3_**), and (**i_1_**–**i_3_**), respectively.

Figure 5 illustrates the microstructure with SMGT alloys aged at 210 °C for different times. The microstructure with SMGT alloys aged for 6 min had nanograins and submicrograins (Figure 5a), while that aged for 27 and 60 min had a random distribution of elongated and equiaxed grains (Figure 5d,g), as shown in the bright-field TEM images. Moreover, as shown in the SAD patterns, it can be observed that the number of grain spots gradually decreased with aging time (Figure 5a,d,g), showing coarsening and growth of the grains were evident with aging time. Numerous precipitates with an ellipsoidal shape can also be observed inside the grain. Corresponding HRTEM images of the microstructure aged for 6, 27, and 60 min are shown in Figure 5b,e,h, respectively. The length of all precipitates for the three aging times ranged from 5 to 8.5 nm, showing that the nanoscale precipitates were smaller than those formed without SMGT alloys. The typical STEM-EDS patterns show that the Cu and Mg elements in the matrix underwent segregated distribution, indicating that S′/S phases were formed during the aging process (Figure 5c–c_3_,f–f_3_,i–i_3_), which is consistent with that observed without SMGT alloys.

**Figure 6:**
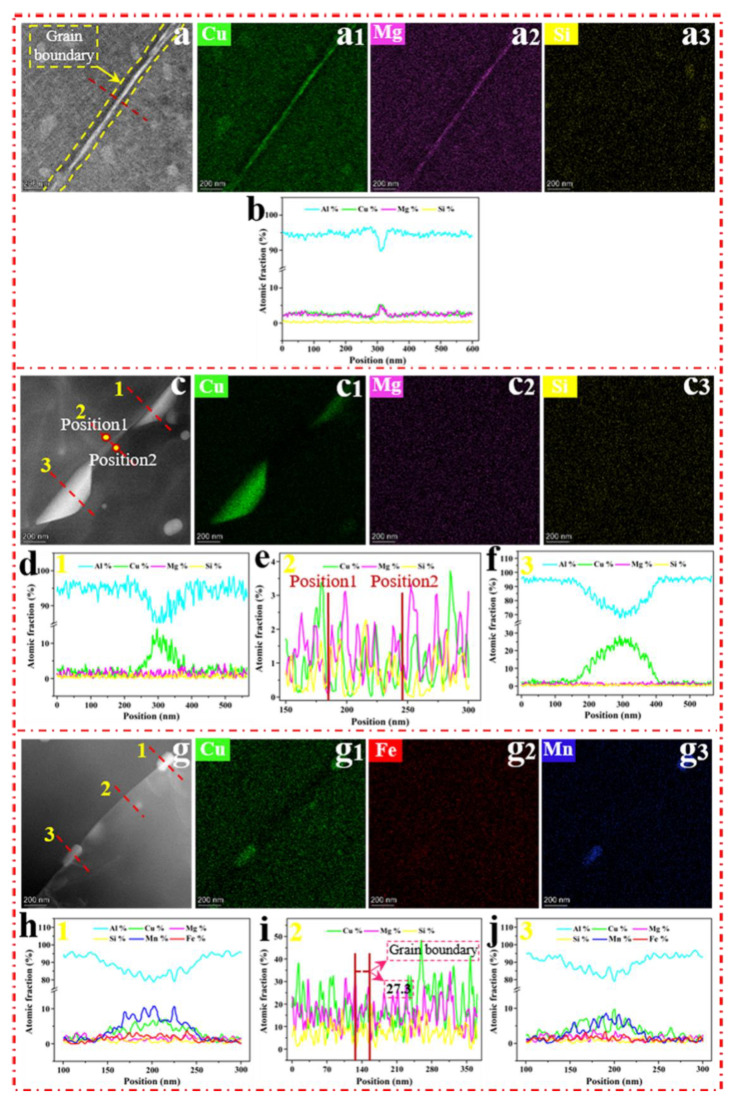
HAADF-STEM images of the grain boundaries without SMGT alloy under 210 °C aging for different times: (**a**) 6 min, (**c**) 27 min, and (**g**) 60 min. The corresponding EDS elemental mappings are shown in (**a_1_**–**a_3_**), (**c_1_**–**c_3_**), and (**g_1_**–**g_3_**), respectively. The HAADF-STEM images show lines across the grain boundary (**a**,**c**,**g**). The corresponding EDS line scan curves are displayed in (**b**), (**d**–**f**) and (**h**–**j**) of (**a**), (**c**) and (**g**), respectively.

Typical HAADF-STEM images of the grain boundaries without SMGT alloys aged at 210 °C for different times are shown in Figure 6. The STEM-EDS pattern analysis for samples aged for 6 min in Figure 6a–a_3_ reveal segregation of the Cu and Mg elements along the grain boundaries. According to the representative EDS line scanning (Figure 6b) across the grain boundary (indicated by the red line in Figure 6a), the atomic fraction of Cu was 5.3%, and the Cu/Mg atomic ratio was close to 1, but no precipitates were observed at the grain boundary. Furthermore, after aging for 27 min, it can be observed that precipitates with irregular shape were formed at the grain boundary, as shown in the STEM image (Figure 6c), EDS maps (Figure 6c_1_–c_3_), and lines (Figure 6d,f). This reveals that the precipitates formed at the grain boundary were Cu-rich precipitates, resulting in a decrease in the Cu concentration at the grain boundary around the Cu-rich precipitates compared to that in the surrounding matrix (Figure 6e). 

Figure 6g displays a STEM image of the morphology of rod-shaped precipitates formed at the grain boundary after aging for 60 min. One can see that the EDS maps (Figure 6g_1_–g_3_) and lines (Figure 6h,j) reveal that the precipitates were Cu- and Mn-rich phases, and the Cu element was depleted at the grain boundary (Figure 6i) from the surrounding precipitates, which were formed along the grain boundary. These phenomena indicate that more Cu-rich precipitates were formed at the grain boundary without SMGT alloys after aging for 27 and 60 min. 

**Figure 7:**
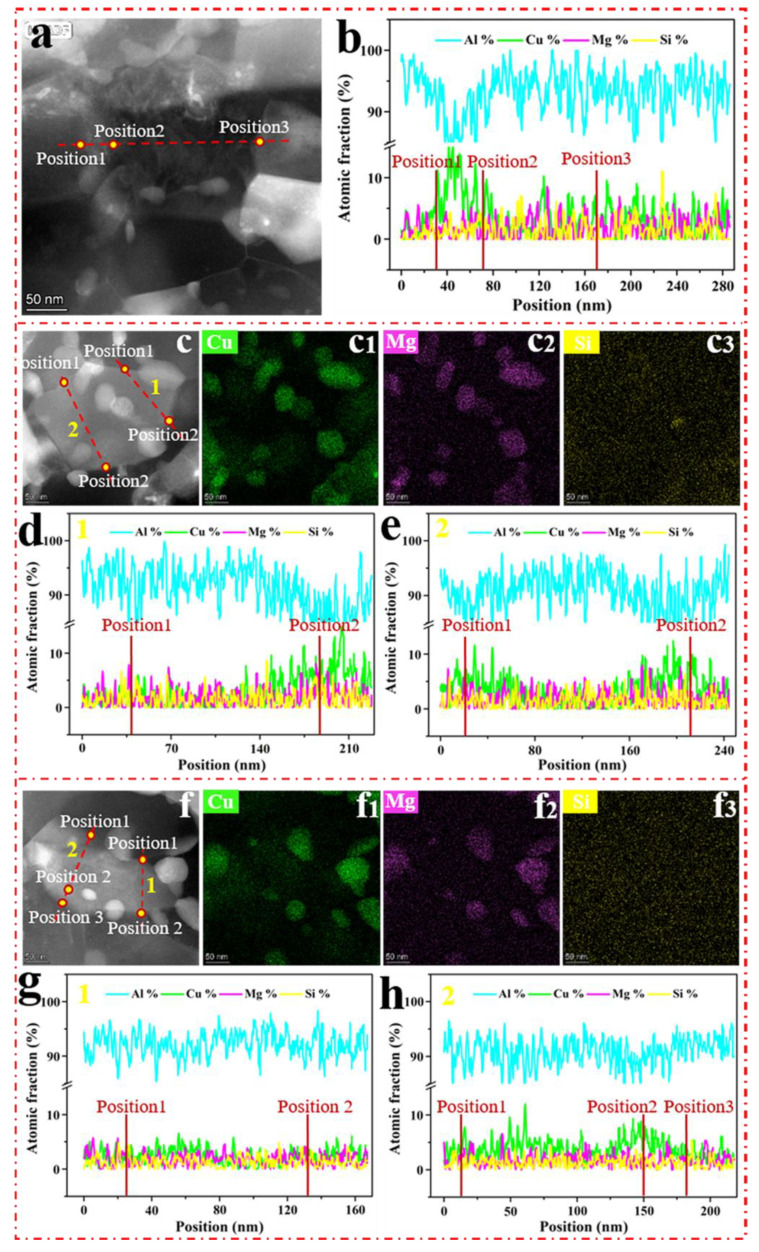
HAADF-STEM images of the grain boundaries with SMGT alloy under 210 °C aging for different times: (**a**) 6 min, (**c**) 27 min, and (**f**) 60 min. EDS elemental mappings are shown in (**c_1_**–**c_3_**) and (**f_1_**–**f_3_**) of (**c**) and (**f**), respectively. The HAADF-STEM images show lines across the grain boundary (**a**,**c**,**f**). The corresponding EDS line scan curves are displayed in (**b**), (**d**,**e**) and (**g**,**h**) of (**a**), (**c**) and (**f**), respectively.

HAADF-STEM images of the grain boundaries with SMGT alloys aged at 210 °C for different times are shown in Figure 7. Figure 7a,b display one example after aging for 6 min, where the concentration profiles of the elements across the grain boundary measured by STEM-EDS line scanning were included (Figure 7b). As shown in Figure 7a, at the grain boundary, some ellipsoidal precipitates were formed, which is inconsistent with that observed without SMGT alloys, where no precipitate was observed after aging for 6 min. This shows that the precipitation behavior was facilitated with SMGT alloys during early aging. In addition, the atomic fraction of Cu was approximately 12.5%, and the Cu/Mg atomic ratio at the grain boundary was approximately 6.8 (Figure 7b), which is larger than the component ratio of the alloy (Cu/Mg atomic ratio was 1.18). This is different to that observed without SMGT alloys, in which the atomic fraction of Cu was 5.3% and the Cu/Mg atomic ratio was close to 1 after aging for 6 min. These results demonstrate that, during early aging, with SMGT alloys, diffusion of Cu atoms from the grain toward the grain boundaries was accelerated when compared to that of Mg atoms, and the diffusion rate of Cu atoms from the grain toward the grain boundaries was much higher than that without SMGT alloys.

STEM images of the ellipsoidal precipitates formed at the grain boundary after aging for 27 and 60 min are displayed in Figure 7c,f, respectively. The EDS maps (Figure 7c_1_–c_3_,f_1_–f_3_) reveal the presence of a large number of Cu- and Mg-rich precipitates, namely, S′/S phases, while for EDS line scanning (Figure 7d,e,g,h) across the grain boundaries (respectively, indicated by the red lines in Figure 7c,f), the Cu/Mg atomic ratio at the grain boundary is approximately 1.8 and 1.9 after aging for 27 and 60 min, respectively. This is close to the component ratio of the alloy, indicating that the diffusion of Cu and Mg atoms from the grain toward the grain boundaries was simultaneous with SMGT alloys. Moreover, a large number of Cu- and Mg-rich precipitates were formed with SMGT alloys, while more Cu-rich precipitates were formed without SMGT alloys after aging for 27 and 60 min, suggesting that the diffusion of Mg atoms from the grain toward the grain boundaries was promoted with SMGT alloys compared to that without SMGT alloys. 

**Figure 8:**
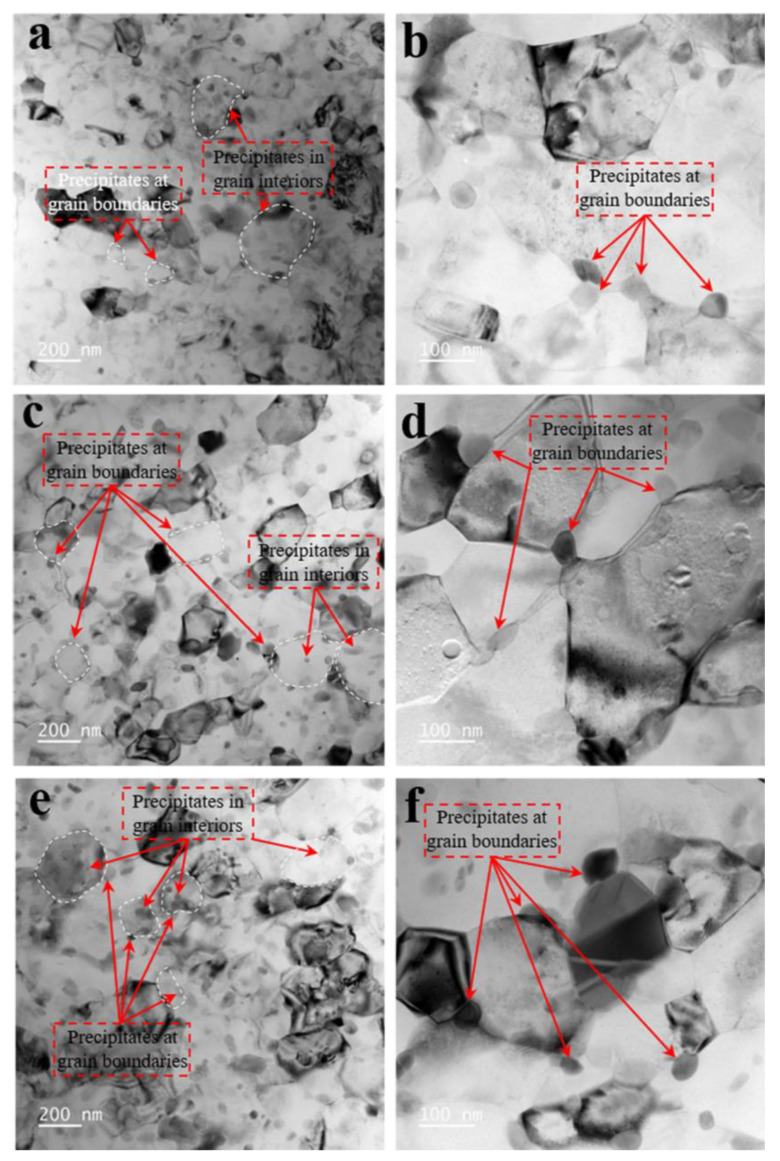
Bright-field images of the evolution of the precipitates with SMGT alloy aged for (**a**,**b**) 6 min, (**c**,**d**) 27 min, and (**e**,**f**) 60 min at 210 °C.

Figure 8 displays the precipitation with SMGT alloys for different aging times at 210 °C. As shown in Figure 8a, after aging for 6 min, numerous precipitates were mainly found inside the grain for submicrograins, while they were mainly found at the grain boundaries for nanograins. This phenomenon is similar to the one reported by Chrominski et al. [28] for Al–Mg–Si alloys. Moreover, fine precipitates were formed at triple points, indicating that solutes from the grain moved toward the grain boundaries and then diffused to triple points, which were the preferred nucleation sites (Figure 8b). These phenomena can also be found in the alloys aged for 27 min (Figure 8c,d) and 60 min (Figure 8e,f). 

### 3.3. Evolution of Precipitate Size

**Figure 9:**
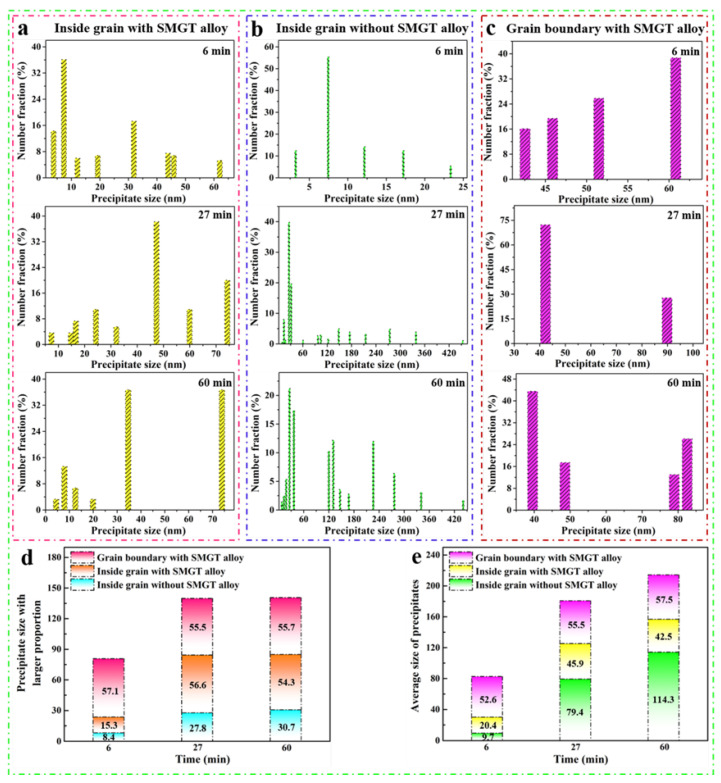
The statistical distribution of precipitate size at 210 °C for different aging times: (**a**) inside grain with SMGT alloy; (**b**) inside grain without SMGT alloy; (**c**) grain boundary with SMGT alloy; (**d**) precipitate size with a larger proportion; (**e**) average size of precipitates with time.

Figure 9a,b show the statistical distribution of the precipitate (S′/S phases) size inside the grain with and without SMGT alloys, respectively. After aging for 6 min, the precipitate size with a larger proportion inside the grain in these two alloys was 15.3 and 8.4 nm, respectively, as shown in Figure 9a,b,d. Moreover, precipitates with a larger size (30–65 nm) were formed inside the grain with SMGT alloys, while this was not observed without SMGT alloys (Figure 9a,b). With the rise in aging time, after aging for 27 min, the precipitate size with a larger proportion in both of these alloys was 56.6 and 27.8 nm (Figure 9a,b,d), respectively, showing that the precipitate size inside the grain increased for these two alloys with increasing aging time. However, it is worth noting that precipitates with a size range of 119–455 nm presented without SMGT alloys (Figure 9b), while this phenomenon was not apparent with SMGT alloys (Figure 9a). With increasing aging time, the precipitate size with a larger proportion in these two alloys was 54.3 and 30.7 nm, respectively, when aged for 60 min (Figure 9a,b,d). The change in size was small in these two alloys when compared to that aged for 27 min. Meanwhile, the proportion of the precipitate with a larger size (119–276 nm) obviously increased without SMGT alloys (Figure 9b), while this was not found with SMGT alloys (Figure 9a). These results indicate that the precipitates formed inside the grain with SMGT alloys had higher thermal stability than those formed without SMGT alloys.

Figure 9c displays the statistical distribution of the precipitate (S′/S phases) size at the grain boundary with SMGT alloys. A certain number of precipitates of a certain size were formed at the grain boundary after aging for 6 min. The precipitate sizes with a larger proportion at the grain boundary were 57.1, 55.5, and 55.7 nm, respectively, after aging for 6, 27, and 60 min (Figure 9c,d), and only a small change appeared with aging time.

Based on quantitative analysis of the distribution of precipitate size, the average size of the precipitates inside the grain and at the grain boundary with and without SMGT alloys was evaluated. As shown in Figure 9e, without SMGT alloys, the average precipitate size value inside the grain was 9.7 nm when aged for 6 min, which increased to 79.4 nm after aging for 27 min, and further increased to 114.3 nm after aging for 60 min, which distinctly increased with increasing aging time. Meanwhile, inside the grain with SMGT alloys, the average sizes were 20.4, 45.9, and 42.5 nm after aging for 6, 27, and 60 min, respectively. The average size of the precipitate inside the grain with SMGT alloys firstly increased to a certain degree and then hardly changed further with increasing aging time. Moreover, the average sizes of the precipitates at the grain boundary with SMGT alloys were 52.6, 55.5, and 57.5 nm, respectively, which changed slightly with increasing aging time. These results show that the thermal stability of the precipitates was much higher with SMGT alloys than that without SMGT alloys.

## 4. Discussion

### 4.1. Microstructure Characteristics during SMGT

**Figure 10:**
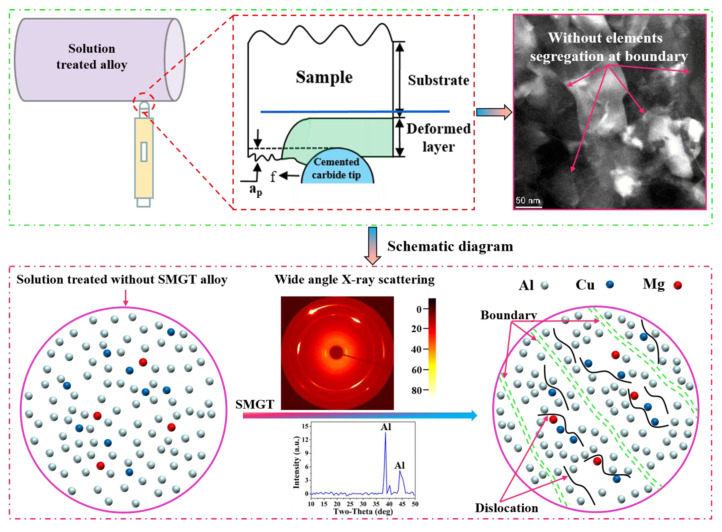
Schematic diagram of the elements’ segregation at the boundary, observed rarely during the SMGT process.

Some studies have indicated that Cu [25,44] and Mg [45] are segregated at the grain boundary after the SPD process before aging. However, in this paper, the STEM-EDS observations (Figure 3d–d_5_) revealed that few elements were segregated at boundary during the SPD process. This phenomenon is contradictory to previous reports, and the difference is attributed to the distinction of the processing route. A schematic diagram is displayed in Figure 10. According to Gray et al. [52], the relationship between the strain rate έ and mobile dislocation density ρ_m_ is given as:

ρ_m =_ έ/(α ν b) (1)

where α is a factor corresponding to ~0.5, ν is the average dislocation velocity, and b is the magnitude of the Burgers vector. Equation (1) indicates that the dislocation density produced during SPD increased with an enhanced strain rate. 

Previous studies have found that the generated dislocation density is approximately ~10^14^ m^−2^ during equal channel angular pressing [27], rolling [25], and high-pressure torsion [24] processes. Meanwhile, in this work, according to wide-angle X-ray scattering measurement, the generated dislocation density was approximately ~10^15^–10^16^ m^−2^ during SMGT processing due to the higher strain rate, which enhanced the order of magnitude when compared to that in the previous processing route. Therefore, solute atoms could be easily attracted and captured by these dislocations and it is hard for them to move to the boundary during SMGT processing, as shown in Figure 10.

### 4.2. Diffusion Characteristics near the Grain Boundary after Aging

**Figure 11:**
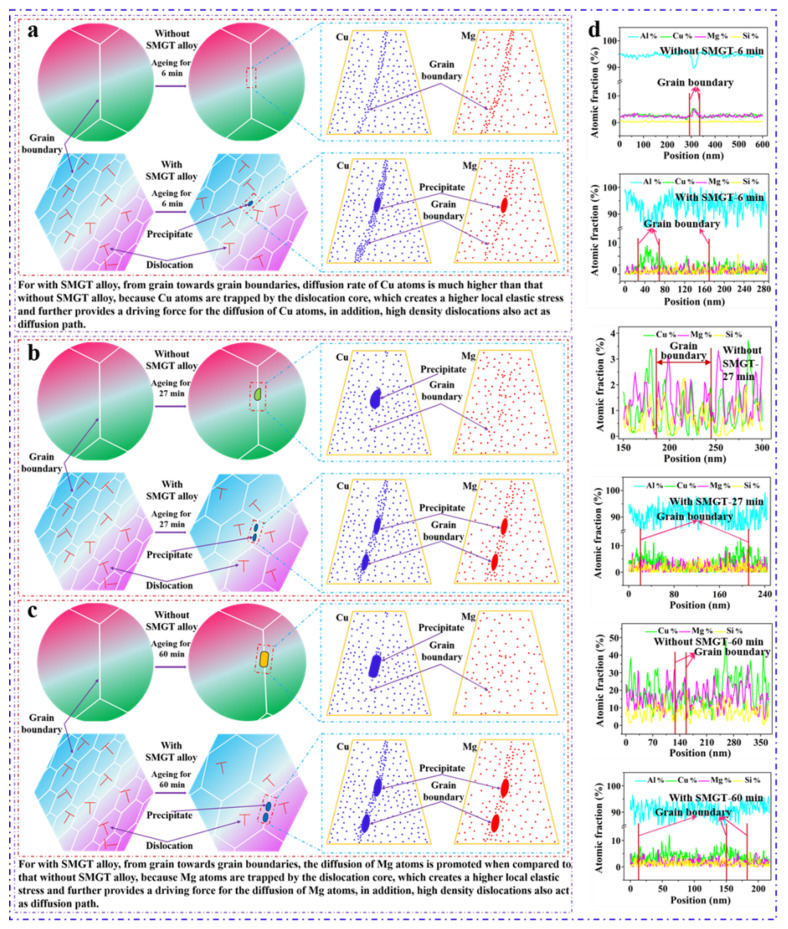
Schematic illustration of the elements’ segregation and precipitation at the grain boundary with and without SMGT alloys under 210 °C aging for (**a**) 6 min, (**b**) 27 min, and (**c**) 60 min. The corresponding STEM-EDS line scan curves are displayed in (**d**).

Figure 11 shows a schematic illustration of the elements’ segregation and precipitation at the grain boundary with and without SMGT alloys under 210 °C aging for 6, 27, and 60 min. This process is closely related to the diffusion of atoms, which requires a certain driving force. For the alloy used in this work, the mass fractions of Cu and Mg were 4.3% and 1.5%, respectively, and the ratio of Cu and Mg atoms was approximately 1.18. Compared to without SMGT alloys, the diffusion of Cu atoms from the grain toward the grain boundaries was remarkably facilitated during early aging, while that of Mg atoms was apparently promoted after aging for 27 and 60 min with SMGT alloys. Because solute atoms (Cu and Mg atoms) were trapped by the dislocation core, which created a higher local elastic stress and further provided a driving force for the diffusion of the solute atoms, high-density dislocations also acted as a diffusion path (Figure 11a–d).

**Figure 12:**
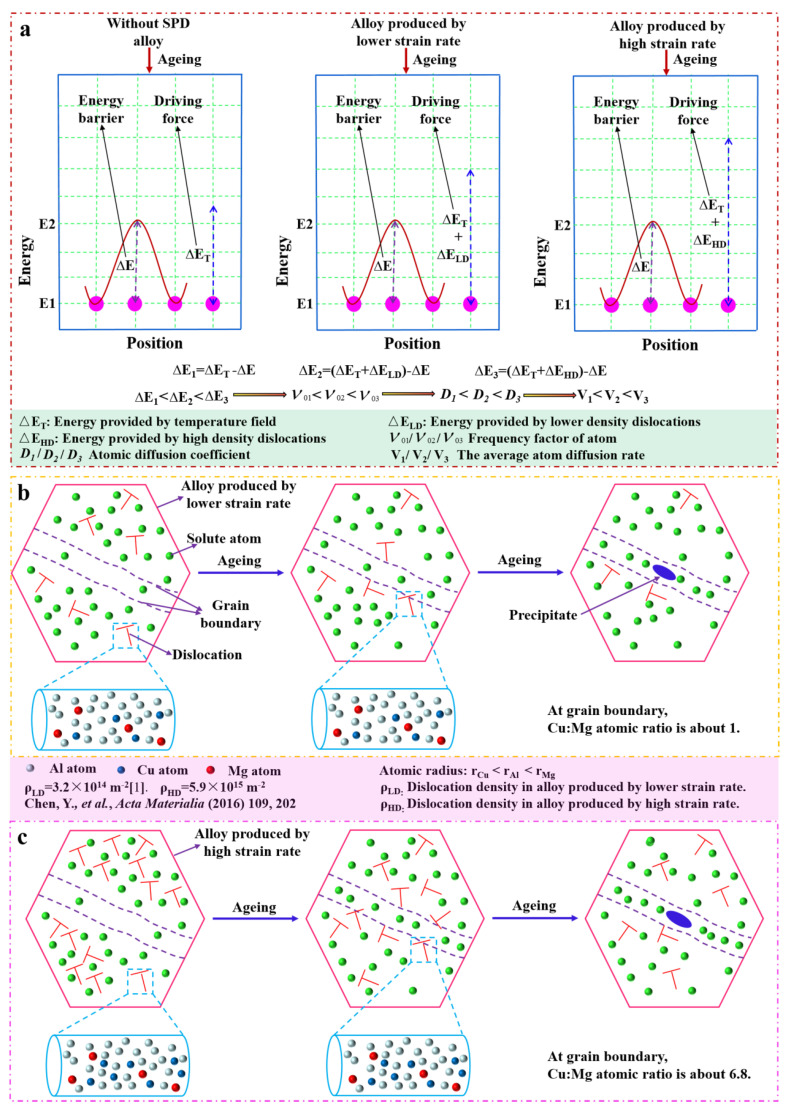
Atomic diffusion in alloys produced with a low or high strain rate during the aging process. Sketch of (**a**) diffusion driving force, diffusion process in alloys produced with a low [24] (**b**) and high (**c**) strain rate.

Figure 12 displays a schematic illustration of the atomic diffusion in the alloys produced by low and high strain rates during the aging process. Based on Equation (1), the dislocation density was relatively lower in the alloys processed with a lower strain rate, while it was higher in the alloys processed with a high strain rate. Gao et al. [24] revealed that the dislocation density was approximately 3.2 × 10^14^ m^−2^ in the UFG Al–Cu–Mg alloy produced with a lower strain rate, while it was approximately 5.9 × 10^15^ m^−2^ in the UFG Al–Cu–Mg alloy produced with a high strain rate in this work. Thus, in these two UFG alloys, the dislocation density was different. Compared to without SPD alloys, the dislocation density apparently increased in these two UFG alloys. Accordingly, the diffusion driving force increased from ∆E_T_ to (∆E_T_ + ∆E_LD_), and finally to (∆E_T_ + ∆E_HD_), ∆E_T_, ∆E_LD_, and ∆E_HD_; energy is, respectively, provided by the temperature field, lower density dislocations, and high-density dislocations (Figure 12a). Based on the atomic diffusion coefficient expression as follows [53]:

(2) $$D\;={\;\alpha }^{2}P\nu_{0}\mathrm{Zexp}\left(\frac{\Delta S_{v}+\Delta S}{k}\right)\exp\left(-\frac{\Delta H_{V}+\Delta H}{\mathrm{kT}}\right)$$

where α is the jump distance of the atoms; P is the probability of a jump in a certain direction; $\nu_{0}$ is the vibration frequency of the atoms; Z is the coordination number of one atom; $\Delta S_{V}$ and ΔS are, respectively, the change in vibrational entropy of the phase transition contacted with the formation, and the displacement of the vacancy; $\Delta H_{V}$ and ΔH are, respectively, the corresponding enthalpy changes; and k is the Boltzmann constant. The increase in the diffusion driving force resulted in an increase in the frequency factor of the atom ($\nu_{01}$ < $\nu_{02}$ < $\nu_{03}$) [54]. According to Equation (2), the corresponding atomic diffusion coefficient increased (*D*_1_ < *D*_2_ < *D*_3_). Eventually, the average diffusion rate of the atoms increased from V_1_ to V_2_ and finally to V_3_.

For the alloy produced with a lower strain rate, the dislocation density was lower, as was the diffusion driving force. Meanwhile, the diffusion was mainly closely related to the composition of the alloy; as a result, the diffusion ratio of the Cu and Mg atoms was approximately 1 (Figure 12b). This is consistent with the results obtained by Gao et al. [24], indicating that the ratio of Cu and Mg at the grain boundary was close to 1 in the Al–Cu–Mg alloy processed with the lower strain rate during aging. Meanwhile, for the alloy produced with the high strain rate, the dislocation density was high, providing many diffusion paths and a high diffusion driving force, the atoms were more active, and atoms with a small radius were more easily moved (Figure 12c). In this work, during early aging, there was not enough energy for atomic diffusion, but the radius of the Cu atoms was approximately 25% smaller than that of the Mg atoms; hence, the diffusion of the Cu atoms from the grain toward the grain boundaries was easier than that of the Mg atoms, and the diffusion ratio of the Cu atoms was larger than that of the Mg atoms. As a result, the Cu/Mg atomic ratio was approximately 6.8 at the grain boundary. However, during later aging, the energy driving force for atomic diffusion was satisfied, the diffusion ratio of the Mg atoms increased, and, therefore, the diffusion ratio of the Cu atoms was slightly larger compared to that of the Mg atoms. Eventually, the Cu/Mg atomic ratio stabilized at approximately 2 at the grain boundary.

### 4.3. Precipitates’ Thermal Stability with SMGT Alloys

The S phase with the lattice parameters a_s_ = 0.400 nm, b_s_ = 0.923 nm, and c_s_ = 0.714 nm, which has a face-centered orthorhombic structure, always forms on {012}_Al_ habit planes and grows mainly along the <100>_Al_ direction [55]. Thus, the thermal stability of precipitates in the <100>_Al_ direction was studied. During the aging process, overall, compared to without SMGT alloys, a much higher number of precipitates (S′/S phases) formed and grew inside the grain with SMGT alloys after aging for 6 min (Figure 9a,b). With increasing aging time (aging for 27 and 60 min), the average size of the precipitates (S′/S phases) formed inside the grain without SMGT alloys was obviously much larger than the average size of those formed inside the grain with SMGT alloys. Namely, the precipitates with SMGT alloys had a higher thermal stability than those formed without SMGT alloys. The difference in thermal stability of the precipitates with and without SMGT alloys can be analyzed in terms of the kinetics of their nucleation and growth, which is closely related to the diffusion of solute atoms.

**Figure 13:**
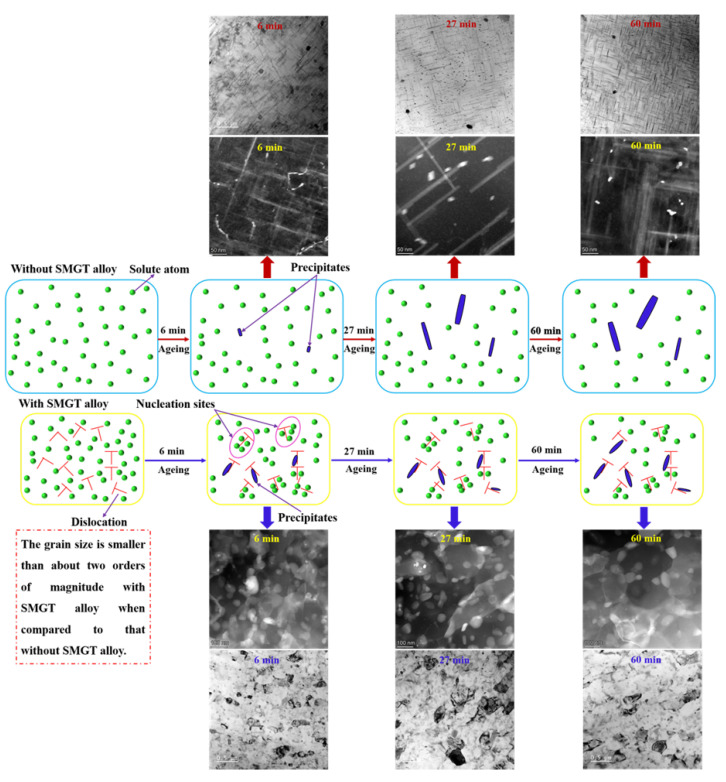
Schematic illustration of the evolution of precipitation inside the grain with and without SMGT alloys under 210 °C aging for 6, 27, and 60 min.

A schematic illustration of the evolution of precipitation inside the grain with and without SMGT alloys at 210 °C aging for different times is displayed in Figure 13. Compared to without SMGT alloys, with SMGT alloys, the diffusion of solute atoms was promoted due to a large number of dislocations serving as the diffusion path. Additionally, more nucleation sites were provided by dislocation with high energy located inside the grain. Therefore, a larger number of precipitates formed inside the grain with SMGT alloys than without SMGT alloys during early aging, as shown in Figure 9a,b and Figure 13. However, more nucleation sites attracted and captured numerous solute atoms, which resulted in supersaturation of the solid solution being strongly lowered inside the grain with SMGT alloys, as shown in Figure 13. Thus, the composition driving force for the further growth of the precipitates inside the grain with SMGT alloys was much smaller than that without SMGT alloys. In addition, the grain size was smaller than approximately two orders of magnitude with SMGT alloys when compared to that without SMGT alloys, which restrained the growth of the precipitates with SMGT alloys. These two factors led to a change in the average size of the precipitates with aging time, being much smaller with SMGT alloys than without (Figure 9e and Figure 13). Hence, the overall thermal stability of the precipitates was much higher with SMGT alloys than without.

## 5. Conclusions

The precipitation behavior in the ultrafine-grained (UFG) Al–Cu–Mg alloy (AA2024) produced by surface mechanical grinding treatment (SMGT) with a high strain rate was investigated. The morphology, size, composition, and distribution of the precipitates were characterized using ex situ transmission electron microscopy (TEM). Conclusions were drawn as follows:

1. The segregation of the elements at the boundary was rarely observed in the SMGT-processed sample before aging, since the solute atoms were hard to segregate at the boundary; segregation was impeded by dislocations produced during the SMGT processing.
2. During early aging, the atomic fraction of Cu at the grain boundary was approximately 12.5% with SMGT alloys, while it was approximately 5.3% without SMGT alloys. Meanwhile, the diffusion rate of the Cu atoms from the grain toward the grain boundaries was accelerated with SMGT alloys, because Cu atoms were trapped by the dislocation core, which created a higher local elastic stress and further provided a driving force for the diffusion of Cu atoms. In addition, high-density dislocations acted as a diffusion path.
3. The combined action in terms of the composition of the alloy, atomic radius, diffusion path, and diffusion driving force provided by high-density dislocations with SMGT alloys led to the Cu/Mg atomic ratio being approximately 6.8 during early aging, while stabilizing at approximately 2 during later aging at the grain boundary.
4. The thermal stability of the precipitates was much higher with SMGT alloys than that without SMGT alloys. During early aging, the average sizes of the precipitates inside the grain were approximately 2 and 10 times larger than those formed after later aging with and without SMGT alloys, respectively, due to more nucleation sites at the dislocation located inside the grain having attracted and captured numerous solute atoms, and the grain size with SMGT alloys being too small to restrain the growth of the precipitates during the aging process.

## Data Availability

Not applicable.

## Supplementary Materials

The following are available online at https://www.mdpi.com/article/10.3390/ma15238687/s1. Figure S1. The solution treated with SMGT alloy: HAADF-STEM image (a) and corresponding EDS elemental maps of copper (a1), magnesium (a2), silicon (a3), iron (a4), and manganese (a5).
