# Supplementary material for: Precipitation Behavior during Aging Operations in an Ultrafine-Grained Al–Cu–Mg Alloy Produced by High-Strain-Rate Processing
Source: Materials (Basel). 2022 Dec 6;15(23):8687. doi: 10.3390/ma15238687 (PMC9737830; doi:10.3390/ma15238687)
Supplement: Supplementary file 1 [file materials-15-08687-s001.zip › materials-1974771-supplementary.pdf]

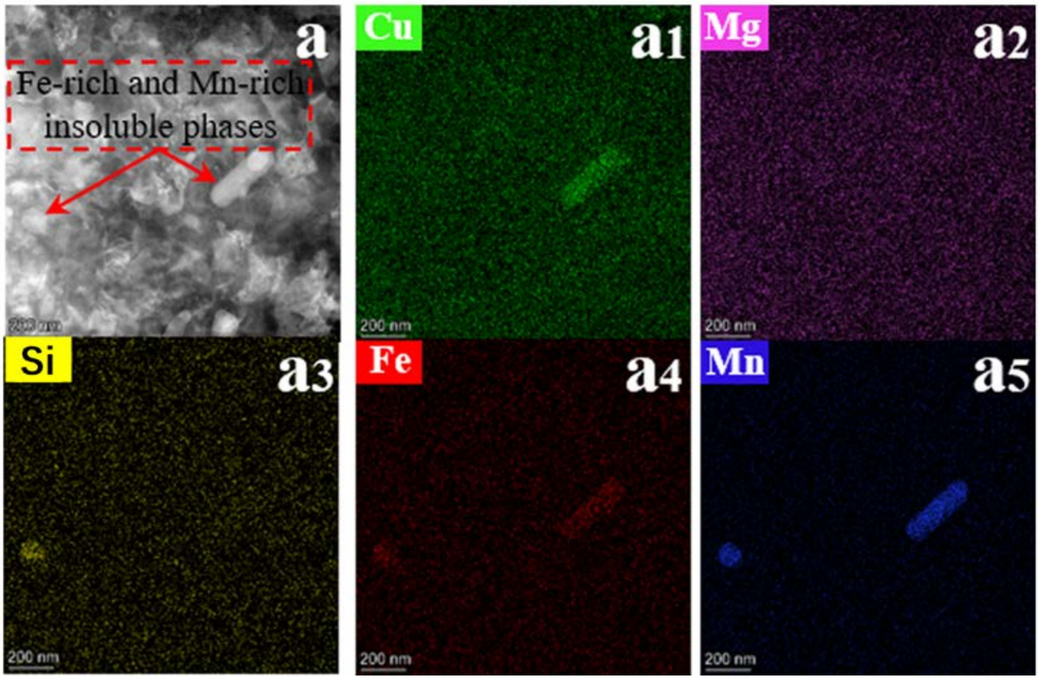

Supplementary Figure S1. The solution treated with SMGT alloy: HAADF-STEM image (a) and corresponding EDS elemental maps of copper (a_1), magnesium (a_2), silicon (a_3), iron (a_4), and manganese (a_5).
